# The Unliveable Misery

**Published:** 1906-09-22

**Authors:** 


### The Hospital

A Journal of

The Medical Sciences and Hospital Administration.

Vol. XL.—No. 1,044. SATURDAY, SEPTEMBER 22, 1906.

### The Unliveable Misery.

Consumption is the cause of more deaths in this country than all the infective fevers put together. In England and Wales 60,000 persons die of it annually—one in every six of the population. It is, moreover, a very fertile cause of pauperism and misery. It is estimated that it costs the City of New York more than four millions yearly, and London, on the same computation, over six million pounds per annum.

Tuberculosis is a home disease, is implanted in the house, develops in the house, and matures in the house. Without the house there probably could be no tuberculosis as a cause of death. It is particularly rampant in the more densely crowded districts, as, for instance, in Stepney, Islington, Southwark, and Lambeth in London. The worst feature is that consumptive people continue to circulate in the most insanitary abodes, and infect in turn each house into which they move. What a blot upon civilisation it is that such places should exist, and that in most cities, least of all in London, nothing is done to disinfect them!

It is a disease implanted in childhood and early life, the breakdown in the vast majority of cases being delayed until later in life. The probabilities are that every inmate in the home of the poor consumptive has tuberculosis in some form or in some stage of the complaint, and constantly act as disseminators of the virulent germs. It is of the utmost importance to impress this on the public mind.

Every case of consumption is derived from an antecedent case, and the infection is almost always conveyed from one patient to another by means of the sputum. Many students of this subject—as, for instance, Sir Patrick Manson—believe that infection is conveyed not only by the sputum, but by the fine vaporised saliva which the patient forcibly blows into the air during a paroxysm of coughing or sneezing. The chief sources of danger in this respect are ordinary hotels, railway trains, buses, and tramcars, churches, theatres, and other public buildings, or any closed place of public resort. It is easy to demonstrate that in the dust on the floors of public buildings, of schoolrooms, of railway carriages, and public conveyances the presence of virulent undiluted, grossly infective tubercle bacilli are present in dangerous colonies. Analysis has been made of many samples taken at random from taverns, railway carriages, schools, hotels, and institutions of various kinds, and with the result that the typical tubercle bacilli were found present in every second example.

On the Continent, in times long past its infectivity was recognised and regulations made for the stringent disinfection with such material as was then at hand of consumptives, their homes, and their wearing apparel. The penalties were severe. Any person found guilty of selling the clothes of consumptives was at one time liable to be sent to the galleys. If a doctor concealed the disease or failed to notify it in those days he was heavily fined, and if the offence was repeated he was sent out of the country. The conveyance in which any consumptive had driven from one place to another could not afterwards be sold either in public markets or by private treaty.

Our organisation for the treatment of consumption has been truly described by Dr. Heron as a mockery and a scandal. The physician knows that his tonic-expectorant is practically useless, and his advice to the patient as to his way of living is utterly beyond the reach of the poor, and frequently even to the well-to-do are mere *placebo*. It is similar with local authorities. Their sterilising and disinfection processes partake more of the nature of a rite and ceremony than a scientific means of disease prevention—on a level with the gun-firing and noisy tom-toms employed in African villages at night to scare away disease demons. This is because suitable scientific disinfectants are negligently applied or altogether ignored by those officials into whose hands the care of the public health has been entrusted.

We must, therefore, until a specific remedy has been discovered, treat the consumptive on purely medical lines. There is great reason to believe, as it is our intention in a future issue of The Hospital to demonstrate, that the new methods, to which attention is drawn in another column, of which the King's Sanatorium may be taken as the highest type, although an expensive model, can be adapted to the needs of this great multitude of consumptive persons in England, and by prompt discovery and treatment of the disease this great wastage of life may be reduced, and those lives rendered happy which otherwise can be nothing else than an unliveable misery.

